# Self-Powered Point-of-Care Device for Galvanic Cell-Based Sample Concentration Measurement

**DOI:** 10.3390/s21082665

**Published:** 2021-04-10

**Authors:** Albert Álvarez-Carulla, Yaiza Montes-Cebrián, Jordi Colomer-Farrarons, Pere Lluís Miribel-Català

**Affiliations:** Discrete-to-Integrated (D2In) Research Group, Department of Electronics and Biomedical Engineering, Faculty of Physics, University of Barcelona (UB), 1 Martí i Franquès St., 08028 Barcelona, Spain; ymontes@ub.edu (Y.M.-C.); jcolomerf@ub.edu (J.C.-F.); peremiribelcatala@ub.edu (P.L.M.-C.)

**Keywords:** self-powered, energy harvesting, fuel cells, point-of-care, supercapacitors

## Abstract

A novel self-powered point-of-care low-power electronics approach for galvanic cell-based sample concentration measurement is presented. The electronic system harvests and senses at the same time from the single cell. The system implements a solution that is suitable in those scenarios where extreme low power is generated from the fuel cell. The proposed approach implements a capacitive-based method to perform a non-linear sweep voltammetry to the cell, but without the need to implement a potentiostat amplifier for that purpose. It provides a digital-user readable result without the need for external non-self-powered devices or instruments compared with other solutions. The system conception was validated for a particular case. The scenario consisted of the measurement of a NaCl solution as the electrolyte, which was related to the conductivity of the sample. The electronic reader continuously measured the current with a transfer function gain of 1.012 V mA^−1^. The overall system exhibited a maximum coefficient of variation of 6.1%, which was an improvement compared with the state-of-the-art. The proof of concept of this electronics system was validated with a maximum power consumption of 5.8 μW using commercial-off-the-self parts.

## 1. Introduction

Our society is facing a great challenge related to health care activities in terms of the carbon footprint and a more patient-centered scenario of application outside hospital premises, where the available resources are limited [1]. In this area, the impact of point-of-care (PoC) devices, combined with telemedia solutions, can reduce the extensive contribution to gas emissions because of unnecessary trips of patients or health workers to care centers, hospitals, etc. Currently, PoC devices are involved in key fields in health care applications [2], with the well-known example of glucose detectors linked with diabetes and their impact in our society [3], where invasive and non-invasive approaches are followed [4]. Remote monitoring of patients (elderly and chronic patients or a population at risk for certain infections) for individualized proactive health care is a great objective that has two main values: (1) the capacity to have quick and continuous control of the patient; and (2) this control does not require the unnecessary trips of patients to the health care center.

Some PoC devices are envisaged for quick responses to disease outbreaks, to minimize their spread, and to prevent the incorrect administration of drugs and therapies, taking into account their carbon footprint impact, as was stated in [1].

A key aspect of PoC devices is the way in which they are powered [5]. Ideally, battery-less PoC devices with no electronics involved represent the best scenario with the greatest environmental impact, using an external resource like a mobile phone, but if quantitative data are demanded by the end-user, in terms of database management, the measurement and processing of the data are mandatory. Therefore, new approaches to self-powered devices are gaining more interest, and among them there are approaches where the sensor acts as the power source.

In the case of wearable devices, some are based on the piezoelectric effect and piezoelectric nanogenerators (PENGs) [6]. This method of energy harvesting depends on the location of the harvester in terms of the mechanical-to-electrical energy conversion and the related human activity, which can be passive or active. There are several state-of-the-art examples. In [7], the authors presented a human motion-based harvester based on zinc oxide (ZnO) nanowires (NWs) and polyvinylidene fluoride (PVDF) operating at low frequencies (<1 Hz) and generating an open-circuit potential (OCP) up to 0.1 V and 10 nA c$m^{-2}$. Reference [8] presented a piezoelectric nanocomposite device for tactile self-powered systems realized by encapsulating ZnO NWs in a parylene C polymer matrix, achieving an OCP up to 10 V and a peak power of 3 μW.

Another very important approach to self-powered sensors is based on the triboelectric effect. In this case, triboelectric nanogenerators (TENGs) have been developed. An extensive review was presented in [6]. In [9], the authors presented an approach for self-powered glucose biosensors based on a 2 cm× 7 cm× 0.08 cm TENG attached to the wearer’s clothes, generating a peak voltage up to 17 V when walking and capable of charging a lithium battery to power the glucose biosensor.

Thermoelectric harvesting based on the Seebeck effect is another main approach to explore. In [10], an extensive study was carried out by analyzing different ambient conditions and placements of thermoelectric generators, including different scenarios, where the thermoelectric energy harvesters (TEHs) were integrated into clothing. A final shirt with a hidden integrated TEH was presented, demonstrating the power generated during different real-life activities, generating a power between 0.5 mW and 5 mW at ambient temperatures from 15 °C to 27 °C. In [11], a proof-of-concept thermoelectric system could generate up to 20.3 μW c$m^{-2}$ with a gradient temperature of 12 ${}^{\circ }C$ and power the microcontroller of a wearable device, and in [12], a battery-less heartbeat detection system-on-a-chip (SoC) was powered by a thermoelectric generator with a temperature gradient of 0.5 °C and a minimum input power of 20 μW.

Among the different approaches to power PoC devices, biofuel cells are of great interest. This is because the output power is usually proportional to the concentration of a metabolite [13]. However, generally, the generated power can be low (lower than the mW level), which represents a constraint for such electronics. Hence, it is interesting to search for a self-powered approach based on fuel-cell PoC devices with the capability to manage low-power energy and to generate a quick response for the end-user for rapid detection, diagnosis, and monitoring purposes.

There are different examples in the case of enzymatic fuel cells [14] and studies on their power output capabilities and lifetime [15], for example to detect the levels of lactate, ascorbate, and glucose. In the work reported in [15], the main issues were related to the maximum power density and available OCP, in the range from 5 μW c$m^{-2}$ to 1.2 mW c$m^{-2}$ and from 540 mV to 1 V, respectively. In some works, the fuel cell in the self-powered approach acted as a sensor. In [16], a glucose self-powered biosensing system was presented in which the cell had the capability to extract a physiological glucose concentration of 5 mM with an OCP of 302.1 mV and 15.98 μW c$m^{-2}$ at 166.3 mV. The linear response of the sensor was obtained thanks to a charge-pump and by monitoring the relationship between the concentration in the sample and the charging frequency for a fixed capacitor. In this case, the result would not be direct data for the end-user. In [17], a self-powered cholesterol biosensor was presented. In this case, the power output was proportional to the cholesterol concentration, and a simple instrument such as a multimeter would be needed to extract the measurement; however, such an interface was not presented.

The next stage is a true full system where the single fuel cell acts as a powering source for the electronics module composed by the front-end interface, control, processing an output interface to extract the measurement. The fuel cell acting also as a sensor [18] will have its limitations compared with those PoC devices based on electrochemical sensors, which is beyond the present work. Some examples can be included. In [19], a self-powered supply-sensing biosensor platform using a biofuel cell was demonstrated powering full electronics, and extracting an output, and pulse rate frequency measurement which are in function of the fructose concentration. As the concentration of fructose is increased, the frequency increases, not following a linear response. As such, it will need calibration for a more reliable reading. In some cases, the extraction from the fuel cell is not reported if power tracking is followed for an efficient power extraction like in [20], where a glucose-based fuel cell has the capability to power a wireless transmission system, at 44 μA and 0.57 V ( 25 μW). In [21] a full system was presented as a plug-and-power PoC platform. A very interesting approach was derived, but in this case, it was disposable or a strip with two elements: a paper-based power source, and the sensor based on a fuel cell, with the capability to have an efficient power tracking, extraction of the measurement and an end-user interface with a power consumption up to 900 μW. In [22], a full electronic solution was presented in terms to cope with the problem to efficiently sense and extract energy from a single fuel cell acting as a power source and as a sensor with a power consumption of 36 μW.

In this paper, we present a new self-powered device solution to cope with the constraint of low energy when a whole self-powered platform is envisaged, combining suitable electronics to have a real output interface for the measurement, in a constrained low-energy scenario, up to 6 μW, and the use of a single energy unit which acts as a sensor and as a powering source at the same time.

The present work presents this proof of concept, and validates this approach for a particular case, experimentally. The system has the capability to manage the powering, sensing and extraction of the measurement just operating with 5.8 μW, and just using commercial-off-the-shelf (COTS) parts. The system has the capability to fix the operating voltage for the fuel cell as a sensor without the use of any potentiostat amplifier, and it generates a control in such a way that no timer or microprocessors it is needed to define the moment to extract the measurement. For this approach, no power tracking is derived because it is envisaged for very low power conditions just to extract the measurement from the cell.

This approach opens new implementations based on application-specific integrated circuit (ASIC) implementations to operate for even lower energies and manage the energy to show or to transmit the data.

## 2. Device Concept and Operation

The device uses a fuel cell as a sensor element and, at the same time, harvests the usually low energy provided by fuel cells commonly used in point-of-care-testing (PoCT) devices applications [23]. It can also operate with batteries intended for sensing, which output characteristics dependent on a parameter to be measured [24]. Both elements intended for sensing, fuel cells and batteries, are referenced as galvanic cells or cells from now on.

The output characteristics of a galvanic cell is usually depicted using its polarization or current vs. voltage (I–V) curves. One of the most common electrochemical method to measure galvanic cells’ polarization curves is linear sweep voltammetry (LSV). The method usually consists of polarizing the cell from its open-circuit potential (OCP) to 0 V. The instrument intended for this electrochemical characterization is the potentiostat. The state of the art of novel galvanic cells’ development is focused on their performance as a powering and sensing elements in order to allow the development of self-powered devices. LSV for different sample concentrations is usually the electrochemical method used to measure this performance. However, while potentiostats provide a way to measure the performance of galvanic cells in order to measure the sample concentration, nowadays, it is not a suitable solution for self-powered portable solutions in scenarios where extreme low power is available from the galvanic cell. Thus, the sample concentration measurement only using the low power provided by a galvanic cell is the challenge that faces the current research in self-powered electronic systems.

In Section 1, different state-of-the-art self-powered approaches are presented. However, these approaches have two major drawbacks: (1) they use a non-self-powered external device or instrument to output the measurement, e.g., oscilloscope, smartphone, etc., and (2) they provide a continuous measurement without a criterion and an indication about when the measurement has ended. We have developed a system that uses a method that overcomes these drawbacks providing a criterion and an indicator for the end of the measurement, and an interface to display the result to the user without external non-self-powered devices involved.

**Figure 1:**
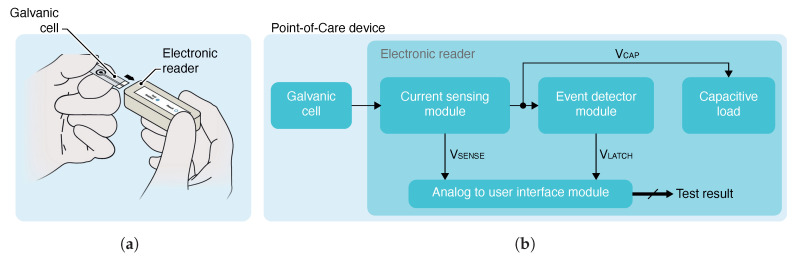
(**a**) Scheme of the application consisting of a disposable test strip and a reusable electronic reader. (**b**) Block diagram of the proposed self-powered point-of-care device.

The application scheme and block diagram of the proposed solution are shown in Figure 1. The system consists of a galvanic cell and an electronic reader. The cell output characteristics depend on a parameter of the cell itself, like the sample concentration used as electrolyte. The electronic reader consists of a current-sensing, event-detector and analog-to-user-interface modules, and a capacitive load.

The solution starts its operation disconnected from the cell or without the sample used as electrolyte deposited, and with its capacitive load initially discharged. The system is intended to start its operation once the cell is plugged or the sample is added. Then, the cell starts to charge the capacitive load. Thus, the polarization voltage applied to the cell corresponds to the charging voltage of the capacitor. This method provides a way to perform a non-linear sweep voltammetry (NLSV) from 0 V to cell OCP. As in conventional LSV, from this method, polarization curves can be measured to distinguish between concentrations. The concentration measurement is usually extracted from current measurement at set polarization voltage $V_{P}$ which usually is selected in terms related to the cell characteristics like sensitivity, repeatability, reproducibility, among others.

The current-sensing module is the responsible for measuring continuously the cell output current during the proposed capacitive load-based NLSV method. The module provides a continuous analog signal $V_{SENSE}$ as an indicator of the current outputted by the cell. In order to provide a result to the user, $V_{SENSE}$ is sourced to an analog-to-user-interface module which converts the analog signal to a user-readable result without needing a non-self-powered external device.

While the current-sensing module provides a continuous measurement, one critical aspect is when the measurement is taken to generate a result and display it to the user. The event-detector module is intended for this task. The event-detector module monitors the charging voltage of the capacitive load $V_{CAP}$. When $V_{CAP}$ reaches a pre-configured desired polarization voltage $V_{P}$, the event detector indicates to the analog-to-user-interface module the occurrence of the event through the signal $V_{LATCH}$. Then, the analog-to-user-interface module captures $V_{SENSE}$ and converts it to a user-readable result and holds its value independent of $V_{SENSE}$ evolution.

## 3. System Implementation

### 3.1. Galvanic Cell

We have implemented a sensing power source based on sodium chloride (NaCl) as authors use in [24]. The presented application in [24] is intended to sense the conductivity in a biological sample to screening cystic fibrosis disease. The NaCl concentrations used are those with an equivalent conductivity in the range of interest for cystic fibrosis screening defined by Diagnostic Sweat Testing Guidelines from the Cystic Fibrosis Foundation [25,26], i.e., NaCl concentrations from 5 to 160 mM.

As a sensing power source, we used a stack of two galvanic cells in series. The cell diagram of each one is

(1) Zn(s)|NaCl(aq)|Cu(s)

where the following electrochemical reaction takes place

(2) $$Zn(s)+2H_{2}O(l)\to Zn(OH){}_{2}(aq)+H{}_{2}(g)$$

The potential provided by each cell corresponds to the Zn(s) oxidation, which has an electromotive force potential $E^{0}$ of 0.761 V [27], and the H^+^ reduction. The Cu(s) electrode acts as an inert electrode in the cell. We used different NaCl solutions with different concentrations as electrolyte. During cell operation, measured potential can vary as a result of electrode polarization [28]. The distance between electrodes was 2.2 cm and the active area of each electrode was 2.5 cm× 2.5 cm. The dimensions of the active areas and the distance between the electrodes are oversized to provide a cell output current in the range of current state-of-the-art galvanic cells with higher current densities and lower sample volume requirements in the range of 15 μL [24]. The development of high current density galvanic cells is outside the scope of this work, and the implemented and used galvanic cell is intended to validate the device with current state-of-the-art galvanic-cells output current ranges.

To implement the cell, we purchased Zn(s) and Cu(s) electrode strips from Pidiscat. For the electrolyte, we have used NaCl solutions with different concentrations using sodium chloride (NaCl, ACS grade, 99.5%) purchased from ITW Reagents and used as received. We have used de-ionized water obtained from a Milli-Q^®^ Advantage A10 water purification system for all experiments. Full-custom electrode holder was 3D printed to control the distance between electrodes and their immersion distance.

### 3.2. Electronic Reader

**Figure 2:**
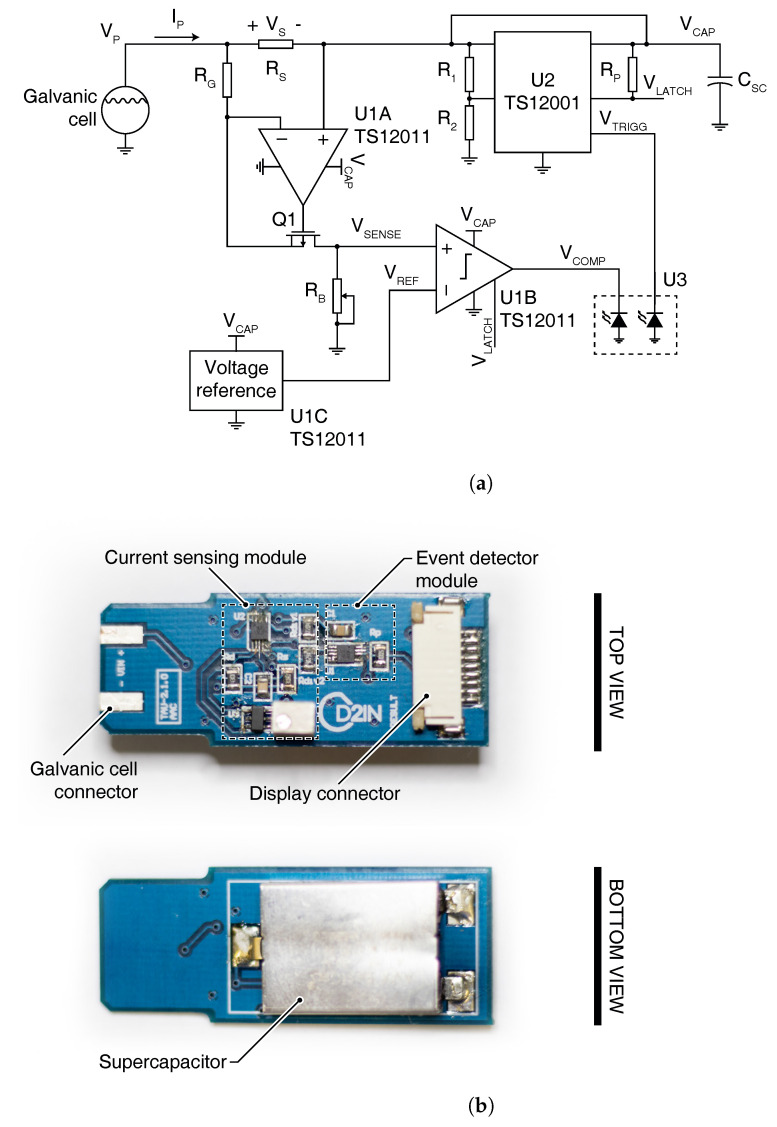
(**a**) Schematic of the implemented self-powered point-of-care device. (**b**) Picture of the implemented self-powered point-of-care device.

We have implemented the device on a 46 mm× 18 mm double-sided PCB. The schematic and picture of the device are shown in Figure 2.

#### 3.2.1. Current-Sensing Module

To sense the cell output current $I_{CELL}$, we have used a high-side current-sensing circuit. A shunt resistor $R_{S}$ is placed in the current path from the cell to the load. Using an operational amplifier (Op Amp), annotated as U1A in Figure 2, and a p-channel MOSFET (PMOS), annotated as Q1 in Figure 2, the voltage drop $V_{S}$ at $R_{S}$, originated by the current flow across the resistor $R_{S}$, is replicated across the resistor $R_{G}$. Thus, a proportional replicated current $I_{G}$ passes through the resistor $R_{B}$ and an output voltage $V_{SENSE}$ is generated. The relationship between the current outputted by the cell and the voltage level of $V_{SENSE}$ is

(3) $$V_{SENSE}=\frac{R_{B}R_{S}}{R_{G}+R_{S}}I_{CELL}$$

The circuit has been previously validated as a reliable current-sensing solution for fuel cell-based systems in [29].

The selection of resistor values for $R_{S}$, $R_{G}$ and $R_{B}$ is discussed in Section 5. The Op Amp available in a TS12011 from Silicon Labs have been used to implement U1A. A PMN70XPX from Nexperia have been used for the PMOS.

#### 3.2.2. Storage Element

We have used a 4.7 mF supercapacitor from AVX as capacitive load and storage element.

#### 3.2.3. Event-Detector Module

The event detector has to detect when its input voltage $V_{CAP}$ passes through the desired polarization voltage $V_{P}$. We have used a well-known circuit that already do this and is usually used in power management units (PMUs) or power converters. The circuit is known as under-voltage lockout (UVLO). We have implemented it using a TS12001 from Silicon Labs, annotated as U2 in Figure 2. When the voltage $V_{CAP}$ reaches the desired polarization voltage $V_{P}$, the signal $V_{TRIGG}$, sourced by the TS12001 pin COUTPP, goes high indicating the end of the measurement. The TS12001 generates a complementary signal $V_{LATCH}$ that we used to latch the result outputted by the analog-to-user-interface module.

#### 3.2.4. Analog-to-User-Interface Module

To convert the analog signal $V_{SENSE}$ to a result readable by the user, we have used one latched comparator with a voltage reference. The circuit operates as a 1 bit flash analog-to-digital converter (ADC) to provide a positive/negative output result indicating to the user if the sample concentration is below or above a concentration threshold. In this work, a concentration threshold of 60 mM has been used following the indications provided by Diagnostic Sweat Testing Guidelines from the Cystic Fibrosis Foundation [25,26]. The signal $V_{SENSE}$ is connected to the positive input of the comparator while the voltage $V_{REF}$ outputted by the voltage reference is connected to the negative input. We have used the latched comparator and voltage reference available in the TS12011 from Silicon Labs. The comparator’s output $V_{COMP}$ toggles normally when the incoming signal $V_{LATCH}$ remains high. When $V_{LATCH}$ goes low, $V_{COMP}$ remains latched holding the result of the measurement. This is one of the main features of the presented solution not requiring a timer or a clock generator, a usually relatively high-power consumption peripherals, to set a controlled instant when the measurement must be taken. $V_{COMP}$, in conjunction with $V_{TRIGG}$, are connected to a low-power electrochromic display, where $V_{TRIGG}$ indicates the end of the measurement and $V_{COMP}$ its result.

## 4. Experimental Setup

For the characterization of the NaCl-based galvanic cell, we have carried out electrochemical experiments for a concentration range with an equivalent conductivity in the range of interest for cystic fibrosis screening defined by Diagnostic Sweat Testing Guidelines from the Cystic Fibrosis Foundation [25,26]. We have used NaCl solutions with a concentration of 5 mM, 30 mM, 60 mM and 160 mM. For a given concentration, we have performed a LSV from cell OCP to 0 V with a scan rate of 10 mV $s^{-1}$. LSV measurements were carried out using a PalmSens4 potentiostat from PalmSens. Fresh solutions and new electrodes were used for each measurement that were performed. All experiments were carried out in a laboratory with controlled environmental conditions.

This characterization serves to validate the construction of the implemented galvanic cell and its performance using a traditional method. However, to select the proper values for the electronic reader passive components, we have also carried out a characterization using the method used by the electronic reader, i.e., a discharged 4.5 mF supercapacitor is connected to the galvanic cell using NaCl solutions with a concentration of 5 mM, 30 mM, 60 mM and 160 mM. Simultaneously, its voltage and output current are logged using a Keysight B2962A source meter unit (SMU).

To characterize the overall device, we have plugged the electronic reader in the galvanic cell with sample concentrations from 5 mM to 160 mM. During the characterization, we have logged analog signals with two Keysight MSOX3034A oscilloscopes in common triggered configuration. We have also characterized its power consumption using the SMU.

## 5. Results

**Figure 3:**
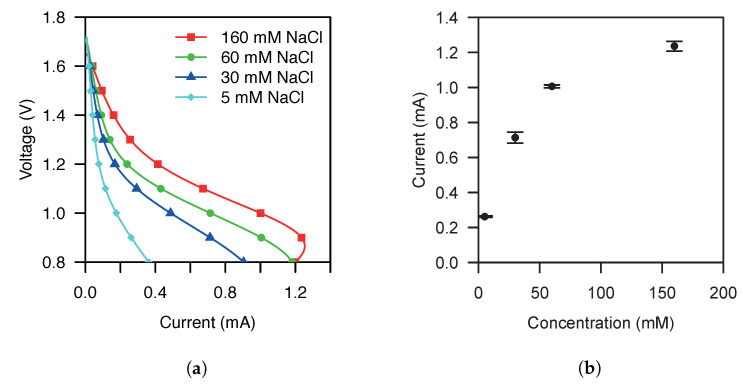
(**a**) Polarization curves of two NaCl-based galvanic cells stacked in series using an opencircuit potential to 0 V linear sweep voltammetry with a scan rate of 10 mVs^−1^ for samples with an increasing conductivity of 5, 30, 60 and 160 mM equiv NaCl. (**b**) Current vs. NaCl sample concentration transfer function extracted for a polarization voltage of 0.9 V from polarization curves of two NaCl-based galvanic cells stacked in series using an open-circuit potential to 0 V linear sweep voltammetry with a scan rate of 10 mVs^−1^ for samples with an increasing conductivity of 5, 30, 60 and 160mM equiv NaCl.

Figure 3 shows the experimental results of an OCP to 0 V LVS for the galvanic cell characterization. The cell exhibits an OCP of 1.715 V and a maximum current range from 0.362 mA to 1.254 mA over the concentration range of interest. In the polarization curves shown in Figure 3a, we can see a maximum sensitivity at 0.9 V. The transfer function of the cell for a $V_{P}$ of 0.9 V is shown in Figure 3b. The measurements of the cell transfer function exhibit a maximum standard deviation of 31.035 μA at 30 mM. Furthermore, at 30 mM, the transfer function exhibits a maximum coefficient of variation of 4.3%.

**Figure 4:**
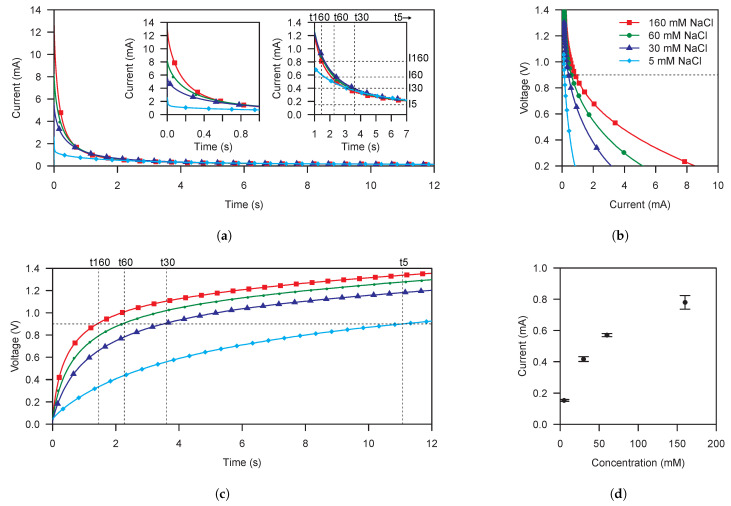
(**a**) Output current transient waveforms of two NaCl-based galvanic cells stacked in series using the 0 V to open-circuit potential capacitor-based non-linear sweep voltammetry for samples with an increasing conductivity of 5, 30, 60 and 160 mM equiv NaCl. The label pairs (*t*5, *I*5), (*t*30, *I*30), (*t*60, *I*60), and (*t*160, *I*160) correspond to the time when a measurement is performed and the corresponding measured current for an increasing conductivity of 5, 30, 60 and 160 mM equiv NaCl, respectively. (**b**) Polarization curves of two NaCl-based galvanic cells stacked in series using the 0 V to open-circuit potential capacitor-based non-linear sweep voltammetry for samples with an increasing conductivity of 5, 30, 60 and 160 mM equiv NaCl. (**c**) Output voltage transient waveforms of two NaCl-based galvanic cells stacked in series using the 0 V to open-circuit potential capacitor-based non-linear sweep voltammetry for samples with an increasing conductivity of 5, 30, 60 and 160 mM equiv NaCl. The label pairs (*t*5, *I*5), (*t*30, *I*30), (*t*60, *I*60), and (*t*160, *I*160) correspond to the time when a measurement is performed and the corresponding measured current for an increasing conductivity of 5, 30, 60 and 160 mM equiv NaCl, respectively. (**d**) Current vs. NaCl sample concentration transfer function extracted for a polarization voltage of 0.9 V from polarization curves of two NaCl-based galvanic cells stacked in series using the 0V to open-circuit potential capacitor-based non-linear sweep voltammetry for samples with an increasing conductivity of 5, 30, 60 and 160 mM equiv NaCl.

Figure 4 shows the results for the cell characterization using the supercapacitor-based method. Figure 4a,c show the output current and voltage of the cell. While Figure 4a reminds us the waveform resulting from a chronoamperometry, it has to be noted that this is not the case as we are performing a NLSV as can be seen in Figure 4c, where the voltage applied is not constant. The voltage at a given time depends on the concentration of the solution and the capacitance of the supercapacitor. Thus, a 0 V to OCP NLSV is applied to the cell.

The inner left graph of Figure 4a shows the current during the first second of the NLSV. Different output current peaks are observed as consequence of the initial short-circuit applied to the cell terminals. In addition, the inner right graph of Figure 4a shows the output current waveforms in the time range where the electronic reader latches the current measurement, i.e.,when the polarization voltage is 0.9 V, as detailed below.

Figure 4b shows the corresponding polarization curves extracted from Figure 4a,c. We can discriminate between the different sample concentrations getting maximum sensitivity in lower voltages. However, the same voltage used to polarize the cell is used to power the electronic reader. Due to that, with the used commercial-off-the-shelf (COTS) parts to implement the electronic reader, a minimum polarization voltage of 0.8V is needed. We have also added 0.1 V to the polarization voltage in order to provide a safe margin to the power voltage of the electronic reader. Figure 4d shows the transfer function obtained for a polarization voltage of 0.9 V. It exhibits a maximum standard deviation of 43.589 μA at 160 mM. Furthermore, at 160 mM, the transfer function exhibits a maximum coefficient of variation of 5.5%.

The characterization of the galvanic cell establishes the constraints to set the values of the passive components of the electronic reader:

1. The maximum current level of 0.780 mA provided by the cell for a $V_{P}$ of 0.9 V over the concentration range of interest sets the value of the shunt resistor $R_{S}$. We have used a value of 30 Ω for $R_{S}$. This value provides a maximum voltage drop $V_{S}$ of 23.4 mV, which corresponds to a 2.6 deviation of $V_{CAP}$ with respect the desired $V_{P}$.
2. The desired polarization voltage $V_{P}$ sets the voltage supply available when the event detector latches the measurement result. Thus, the available output voltage range is also defined being 0 to 0.9 V. To provide a safe headroom margin of 0.1 V, we have maximized the response of the current-sensing module from 0 to 0.8 V. We have used a resistor value of 4.3 kΩ for $R_{G}$, and we have trimmed a potentiometer $R_{B}$ to set, following (3), an overall current sensing module gain of 1.012 V m$A^{-1}$.
3. We have used resistors values of 2.2 and 4.02 MΩ for $R_{1}$ and $R_{2}$ as indicated by the TS12001 datasheet in order to set the detection voltage to 0.9 V.

**Figure 5:**
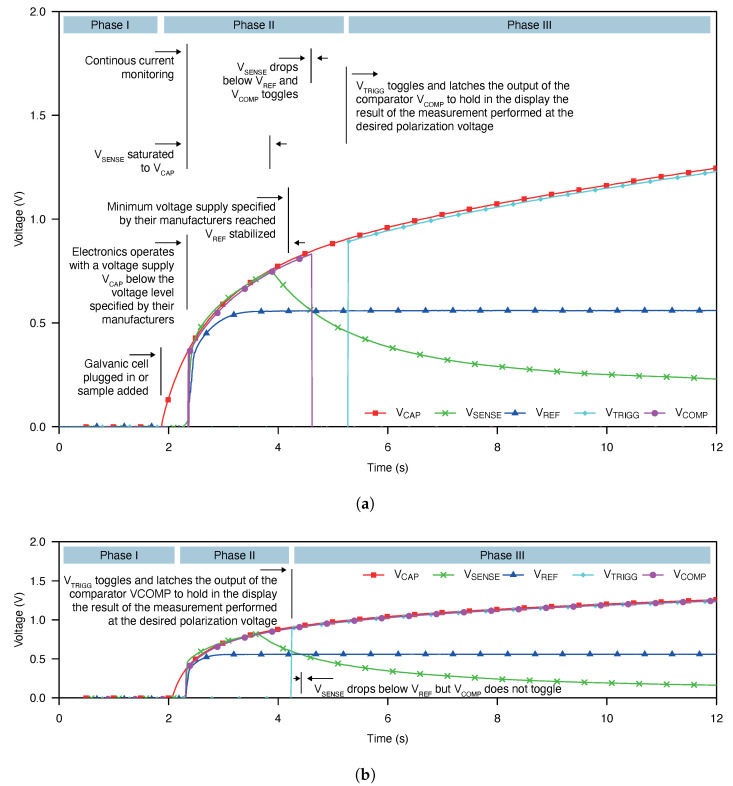
(**a**) Transient waveforms of the signals involved during the measurement of a sample concentration below the threshold concentration using the implemented self-powered point-of-care device. (**b**) Transient waveforms of the signals involved during the measurement of a sample concentration above the threshold concentration using the implemented self-powered point-of-care device.

Figure 5 shows the transient waveform obtained for an under-threshold and an over-threshold sample concentration. Similar waveforms with different measurement times for the other concentrations were obtained. Only two cases are presented, and they are intended to depict the different phases during the measurement. Figure 5 also shows, in detail, the different phases and events during the measurement.

The process consists of three phases. During the first phase, the cell is not plugged to the electronic reader yet and no measurement is performed. Once it is plugged, the process enters phase II. In phase II, the continuous measurement of the cell output current starts. The polarization voltage remains below 0.9 V and the comparator output operates normally. Once a polarization voltage of 0.9 V is reached, the event detector latches the comparator output and holds the measurement result, entering phase III.

In Figure 5a, we can notice how $V_{SENSE}$ drops below the threshold defined by $V_{REF}$ during phase II occasioning comparator output to go low before the event detector latches the measurement result. Alternatively, Figure 5b shows how $V_{SENSE}$ remains above $V_{REF}$ during phase II, causing the comparator output to go high. When the measurement enters phase III, the result is latched remaining in high state even if $V_{SENSE}$ goes under $V_{REF}$.

**Figure 6:**
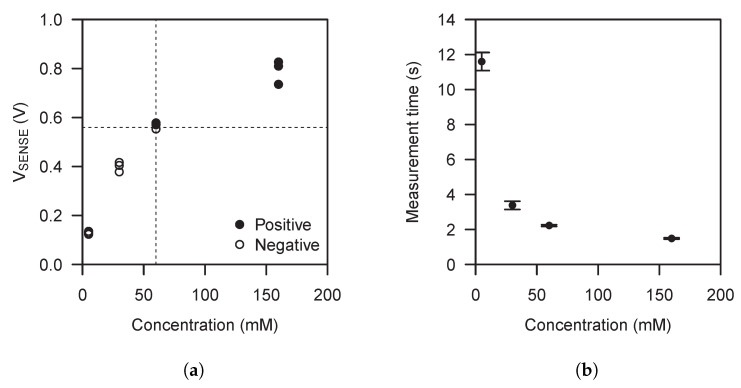
(**a**) Transfer function and comparator output results using the implemented self-powered point-of-care device configured for sample concentration discrimination above and below 60 mM. (**b**) Time needed by the implemented self-powered point-of-care device to perform the measurement for different sample concentrations.

Figure 6a shows the transfer function of the complete system, cell and electronic reader, using NaCl solutions with a concentration of 5 mM, 30 mM, 60 mM and 160 mM. It can be seen how the transfer function output expands along all the available output voltage range over the concentration range of interest. The system exhibits a maximum standard deviation of 49 mV at 160 mM. At 5 mM, the system exhibits a maximum coefficient of variation of 6.1%. Figure 6a also shows the comparator output. We can see how the electronic reader is able to discriminate solution concentrations over or under 60 mM. The comparator output results are normally used for galvanic cell performance evaluation, like repeatability and reproducibility, which is outside the scope of this work, but the results serve us to see how the electronic reader provides a method to discriminate a parameter related to the attached galvanic cell like, in this case, sample concentration.

In all experiments, a time between when the cell is plugged and when the electronic reader event detector latches the measurement result, indicating the end of the measurement, is needed. Figure 6b shows these measurement times. As can be seen, the measurement time mainly depends on solution concentrations due to the different power levels delivered by the different sample concentrations.

Finally, during all experiments, the measured electronic reader power consumption remains below 5.8 μW.

## 6. Conclusions

We have presented a novel electrochemical characterization approach intended for self-powered applications. The system consists of a galvanic cell and an electronic reader, and allows us to characterize a parameter related to the galvanic cell. In this work, we have used the solution to measure the concentration of the sample used as electrolyte in the galvanic cell. The solution performs a non-linear sweep voltammetry to the galvanic cell by means of an initially discharged supercapacitor attached to the cell as a capacitive load.

We have adapted the system to operate with a NaCl-based galvanic cell in order to provide an application-specific solution proposal to cystic fibrosis screening, like in [24]. We have implemented a custom galvanic cell using Zn as anode, Cu as cathode and a NaCl solution as electrolyte. For NaCl concentrations from 5 to 160 mM, with an equivalent conductivity in the range of interest for cystic fibrosis screening defined by Diagnostic Sweat Testing Guidelines from the Cystic Fibrosis Foundation [25,26], a maximum current of 0.780 mA is achieved with a maximum coefficient of variation of 5.5% at a polarization voltage of 0.9 V using the supercapacitor-based method.

Once the cell is plugged, the electronic reader starts current-monitoring. When event detector module latches the measurement result at a polarization voltage of 0.9 V, the current-sensing module exhibits a transfer function with a gain of 1.012 V m$A^{-1}$, an output range expanded from 0 to 0.8 V and a maximum coefficient of variation of 6.1%. The shunt-resistor-based current-sensing technique used generates a maximum polarization voltage deviation of 2.6%.

The analog-to-user-interface module provides a simple conversion from the analog signal provided by the current-sensing module to a electrochromic display that holds and displays the result to the user. Thus, the use of relatively high-power consumption peripherals like a timer or a clock generator is avoided.

All this is achieved with COTS parts and a maximum power consumption of 5.8 μW. In addition, the power extracted from the cell for its polarization is not wasted and remains stored in the supercapacitor to increase the display autonomy or, in future research, increase the functionality of the device.

However, the solution has two drawbacks that need to be worked on. The first is in relation to the minimum operating voltage. Because it is made with commercial-off-the-shelf components, the minimum operating voltage is 0.8 V according to the specifications of the different manufacturers. This forces us to use a stack of more than one fuel cell in series to increase the overall output voltage. This incurs a higher cost due to the cell implementation, and a larger sample volume for the same current density. To deal with this, the next step in our research is to integrate the system into an application specific integrated circuit that will allow us to lower both the minimum operating voltage of the system and its power consumption so that we do not have to use a fuel cell stack. The second fact that, depending on the application, can be considered a drawback is that continuous monitoring is not possible. However, this is due to the very nature of the application, which is designed to provide an instant measurement. Once the measurement is finished, the result is latched. This makes this solution as it stands not suitable for continuous monitoring.

The cost-effective implementation of the device, its low complexity, and low power operation make this solution suitable for self-powered solutions where a parameter related to a galvanic cell must be measured, like in PoC applications. Furthermore, the low complexity of the device, mainly consisting of passive components, allows the solution to be translated into other eco-friendly state-of-the-art technologies where different materials are used to implement electronic circuits, like paper or molded interconnect devices (MID) [30].

## 7. Patents

From the work reported in this manuscript, a resulting patent titled “Self-Powered Device and Method for Measuring a Parameter of a Sensing Power Cell” has been applied to the European Patent Office (EPO) with the application number EP20383114.4 [31].

## Abbreviations

The following abbreviations are used in this manuscript:

ADC	analog-to-digital converter
ASIC	application-specific integrated circuit
ASSURED	affordable, sensitive, specific, user-friendly, rapid, equipment-free, delivered
COTS	commercial-off-the-shelf
I-V	current vs. voltage
LSV	linear sweep voltammetry
MID	molded interconnect device
NLSV	non-linear sweep voltammetry
NW	nanowire
OCP	open-circuit potential
Op Amp	operational amplifier
PCB	printed circuit board
PENG	piezoelectric nanogenerator
PMOS	p-channel metal-oxide-semiconductor field-effect transistor
PMU	power management unit
PoC	point-of-care
PoCT	point-of-care testing
PVDF	polyvinylidene fluoride
SMU	source meter unit
SoC	system on a chip
TEH	thermoelectric energy harvester
TENG	triboelectric nanogenerator
UVLO	under-voltage lockout
V-I	voltage vs. current
